# Matrix Metalloproteinase-9 Gene Polymorphism and Its Methylation in Stroke Patients

**DOI:** 10.21315/mjms2021.28.6.4

**Published:** 2021-12-22

**Authors:** Omkar Kalidasrao CHOUDHARI, Anita RANI, Geeta KAMPANI, Charanjeet KAUR, Ananya SENGUPTA

**Affiliations:** Vardhman Mahavir Medical College and Safdarjung Hospital, New Delhi, India

**Keywords:** stroke, MMP, ischaemic, MMP polymorphism, MMP-9 methylation, epigenetics

## Abstract

**Background:**

Genetic and environmental factors, along with hypertension, diabetes mellitus and smoking cause accelerated atherosclerosis and, eventually, stroke. Matrix metalloproteinase-9 (MMP-9) are inflammatory mediators of the endoproteinase family, and their polymorphism and methylation are associated with the development of atherosclerosis and stroke. This study explores this association in the Indian population.

**Objective:**

To study the association of MMP gene polymorphism and methylation with the risk of stroke.

**Methods:**

A case-control study was conducted on 100 admitted patients (both genders) diagnosed with ischaemic stroke. Another 100 healthy subjects, not suffering from any chronic illness or stroke, were taken as controls. All participants were genotyped for rs3918242 (MMP-9) by polymerase chain reaction (PCR) and restriction fragment length polymorphism. Methylation of the MMP-9 gene-promoter region was assessed by methylation-specific PCR.

**Results:**

The case (mean age = 61.3 ± 7.36 years old) and control (mean age = 60.68 ± 7.1 years old) groups were age-matched. Among cases, 61 patients were smokers, 55 were diabetic and 53 were hypertensive. A significant risk of ischaemic stroke was associated with the CT genotype (adjusted odds ratio [aOR] = 7.09; *P* < 0.001), TT genotype (aOR = 19.75; *P* < 0.001) and T allele (aOR = 10.71; *P* < 0.001). MMP-9 methylation decreased the risk of stroke (aOR = 0.23; *P* < 0.001).

**Conclusion:**

MMP-9 gene-1562C/T polymorphism (SNP rs3918242) (single-nucleotide polymorphism [SNP] rs3918242) is a potential marker to predict ischaemic stroke and constitutes a significant proportion of the general population. Its polymorphism predisposes to ischaemic stroke, while its methylation is protective.

## Introduction

Stroke is a frequent cause of emergency hospital admissions and often leads to significant disability and mortality (1). The incidence rate of stroke in India is 119–145/100,000 persons-years, with a prevalence rate of 84–262/100,000 cases in the rural and 334–424/100,000 cases in the urban population (2). It is one of the most common diseases requiring prolonged rehabilitation facilities, resulting in a significant economic burden on the health system (3, 4). Stroke is defined by the World Health Organization (WHO) as a sudden onset focal (occasionally global) neurological impairment, presumably of vascular origin, lasting more than 24 h or leading to death (5). The aetiology of stroke is multifactorial, consisting of uncontrolled hypertension, long-standing diabetes mellitus, systemic inflammation and smoking, along with other genetic and environmental factors (6–11).

Many genetic polymorphisms have been identified in the pathogenesis of stroke which is responsible for accelerated atherosclerosis, increased propensity for rupture of the atherosclerotic plaque, subsequent emboli formation and cerebral hypo-perfusion; however, the underlying causative complex molecular mechanisms are yet to be explored (12, 13). Matrix metalloproteinase (MMP) is a group of inflammatory mediators of zinc-containing endo-proteinases responsible for accelerated atherosclerosis. Their role in destabilising the atherosclerotic plaque, which results in the formation of an embolus and a subsequent stroke, is an established phenomenon (13, 14). The MMP gene has a pivotal role in the etiopathogenesis of stroke — MMP polymorphism has been associated with an increased risk of ischaemic stroke and potential disruption of the blood-brain barrier (BBB). This enables MMP-mediated inflammatory cytokines to enter the brain microenvironment and cause haemorrhagic transformation (15, 16). The MMP-9 gene is known to be associated with atherosclerotic plaque instability and the MMP-9 gene-1562C/T polymorphism (single-nucleotide polymorphism [SNP] rs3918242) leads to an increased risk of ischaemic stroke; however, there are some contradictory results as well (17–20). Most of these studies are belonged to developed countries and there is a dearth of literature exploring this polymorphism in the Indian scenario.

Deoxyribose nucleic acid (DNA)-methylation is the most common epigenetic modification and such changes are inherited without alteration in the primary gene sequence (21–22). While low-level MMP-methylation is associated with an increased risk of ischaemic stroke, studies revealing the association between global hypomethylation and the risk for stroke are also scarce with contradictory results (23–24). Thus, this study aims to fulfil this gap in the literature to address the paucity of studies in the Indian population and explore the probability of genetic variability leading to stroke.

## Methods

A case-control study was conducted at the Department of Biochemistry and Internal Medicine, Vardhman Mahavir Medical College and Safdarjung Hospital, New Delhi, India from April 2019 to June 2020. A hundred patients (both genders) diagnosed with ischaemic stroke on computed tomography (CT) scan or magnetic resonance imaging (MRI) as cases and 100 healthy subjects (not suffering from any chronic illness) as controls were recruited for the study using simple random sampling. Matching of study subjects was done based on association and not on proportion. The sample size was calculated based on the results of a previous study (18) which observed that MMP-9 gene polymorphism in the CC genotype was 14.33% in cases and 2.99% in controls. Using these reference values and with 80% power of the study at a 5% level of significance, the minimum required sample size was estimated as 93 patients in each group. Detailed history and general physical examination were recorded for all patients including colour Doppler of neck.

Patients diagnosed with acute ischaemic stroke, defined by the WHO as the presence of rapidly developed clinical signs of focal or global disturbance of cerebral functions lasting more than 24 h with no apparent cause other than of vascular origin and proven by a CT scan or MRI, were included in the study if they agreed to participate. Patients suffering from myocardial infarction or unstable angina, severe heart failure, atrial fibrillation, aortic dissection, deep coma, resistant hypertension, apart from those receiving thrombolytic therapy and lipid-lowering drugs, were excluded from the study.

The Krishgen DNA blood kit, India was used for DNA extraction. Primers were synthesised by Eurofins Scientific, India. The serum used to estimate biochemical profile was processed using Siemens’ ADVIA, Germany, a fully-automated clinical chemistry analyser. The DNA-bisulphite conversion for methylation analysis was done using the Epigentek kit (USA). DNA concentration and purity were checked by a spectrophotometer. Genotyping was done by polymerase chain reaction–restriction fragment length polymorphism (PCR-RFLP) and restriction digestion. For genotyping of SNP rs3918242, the following sequence was used — sense primer 5-GCCTGGCACATAGTAGGCCC-3′; antisense primer 5′-CTTCCTAGCCAGCCGGCATC-3′. A 435 bp fragment was amplified using the aforementioned primers (17). The DNA amplification was done by PCR using template DNA (1 μL), forward primer (0.5 μL), reverse primer (0.5 μL), master mix (10 μL) and nuclease-free water (8 μL). The following PCR conditions were used — initial denaturation at 95 °C for 5 min, followed by 30 cycles of denaturation at 95 °C for 30 sec, annealing at 56.2 °C for 30 sec, extension at 72 °C for 30 sec, final extension at 72 °C for 5 min and was then kept at 4 °C for temporary storage. The restriction endonuclease, SphI, enzyme for digesting the amplicon was identified using an NEB cutter v2.0 (New England Bio Labs Inc.). Overnight digestion by SphI at 37 ^o^C produced bands using PCR mix (10 μL), 10× buffer-B (2 μL), enzyme (1 μL), and nuclease-free water (18 μL). Electrophoresis was performed with 2% agarose gel, the 435 bp position was ascertained if a homozygous allele presented for wild type, the 247 bp and 188 bp positions if it was polymorphic, and the 435 bp, 247 bp and 188 bp positions if the heterozygous allele presented for both wild and polymorphic types.

## Methylation-Specific PCR (MSP)

The primers used for MMP-9 gene-promoter region methylation included – forward: 5′-GAAGTTCGAAATTAGTTTGGTTAAC-3′, and reverse: 5′-TCCCGAATAACTAATATTATAAACGTA-3′; while for the MMP-9 non-methylated region, the primers used were — forward: 5′-AGTTTGAAATTAGTTTGGTTAATGT-3′, and reverse: 5′CCTCCCAAATAACTAATATTATAAACATA-3′. Primers were designed using a methprimer available on www.urogene.org/methprimer. Bisulphite conversion of DNA was done using the Epigentek kit via a thermocycler in the following steps. The DNA was kept at 95 ^o^C for 4 min, at 65 ^o^C for 30 min, then again at 95 ^o^C for 4 min and 65 ^o^C for another 30 min, followed by another 4 min at 95 ^o^C and for 60 min at 65 ^o^C. The modified DNA so obtained was subjected to PCR under the following conditions — initial denaturation at 95 ^o^C for 5 min; afterwards, 40 cycles of denaturation at 95 ^o^C for 30 sec, annealing at 58.7 ^o^C for 30 sec, extension at 72 ^o^C for 30 sec, final extension at 72 ^o^C for 5 min and temporary storage at 4 ^o^C, were performed. The obtained products were loaded (7 μL) on 2% agarose gels with ethidium bromide and subjected to electrophoresis and the DNA bands were visualised via a gel documentation system. If the product of 108 bp was present in the set of methylated-primer so obtained in either of the subjects, it indicated that methylation was present in the promoter region of the MMP-9 gene. Alternately, if the 108 bp product was present in the set of unmethylated primers, methylation was absent in the promoter region of the MMP-9 gene.

## Statistical Analysis

Statistical analyses were performed using SPSS version 21 (IBM Inc., Chicago, IL). Mean and standard deviation (SD) were calculated for demographic and basic clinical data. Student’s *t*-test was performed for continuous variables, while dichotomous variables were analysed using the Chi-squared (χ^2^) or Fisher’s test. Also, the Hardy–Weinberg equilibrium was assessed by Chi-squared test. Lastly, binary logistic regression was used to obtain odds ratio (OR) to assess the strength of association between polymorphism of the MMP-9 gene and its methylation in ischaemic stroke. A *P*-value of < 0.05 was considered statistically significant.

## Results

The cases consisted of 100 patients mean age = 61.3 (7.36) years old diagnosed with a stroke, of which 62 were males and 38 were females. The control group comprised of 100 age-matched patients mean age = 60.68 (7.1) years old; *n* = 66 males and *n* = 34 females visiting the medicine outpatient department for problems other than stroke. The two groups were statistically similar in terms of age (*P* > 0.05). Among the cases, 61% were smokers, 55% were diabetic and 53% were hypertensive; and there was no statistically significant difference between the groups regarding the occurrence of hypertension, diabetes and smokers (*P* > 0.05).

The level of biochemical parameters of the case and control subjects as compared using *t*-test are shown in Table 1. Among the cases, 61% had normal sinus rhythm (NSR) on electrocardiography (ECG), followed by atrial fibrillation in 22% of subjects. T-inversion in anterior leads was seen in 5% of the cases and T-inversion in the lateral and inferior leads was seen in 3% of subjects in each group.

Imaging of the head via CT scan or MRI in the case subjects revealed that the right middle cerebral artery territory infarct was the most common entity (14%), followed by the left frontoparietal region infarct, comprising 12% of the cases. Right ganglio-capsular infarcts and left middle cerebral artery infarcts were found in 11% and 10% of the case subjects, respectively, while infarcts affecting the corona radiata were seen in 7% of the cases.

Colour Doppler imaging of the neck to evaluate cerebral blood flow did not reveal any abnormality in 37% of cases; whereas, variable amounts of plaque formation were seen in both left and right carotid arteries in 40% of the stroke patients. About 14% of patients had near-total occlusion of the distal right carotid artery (a branch of the internal carotid artery), while 9% of the stroke patients had 30% stenosis in the right carotid artery.

### Genotype Frequencies

Single-nucleotide polymorphism (SNP) analysis of the MMP-9 gene was performed by PCR-RFLP for SNP rs3918242. The frequency distribution of genotypes and alleles are shown in Table 2. Out of the 100 cases of MMP-9 gene SNPs, the analysis for rs3918242 (C→T) showed a genotypic distribution of 26% with the CC genotypes, 35% with the CT genotypes and 39% having the TT genotypes. Whereas, among the 100 controls, 79% showed CC genotypes, 15% showed CT genotypes and 6% showed the TT genotypes. There was a statistically significant difference in the genotypic distribution of SNP rs3918242 polymorphism between the patients and controls. Further, the Chi-squared test was used to assess the deviation of genotype distribution, among cases and controls from Hardy-Weinberg equilibrium. The prevalence of the MMP-9 genotypes in both groups was not within the range of the Hardy-Weinberg equilibrium (χ^2^ = 58.95, df = 2, *P* < 0.001) as shown in Table 3.

**Figure 1:**
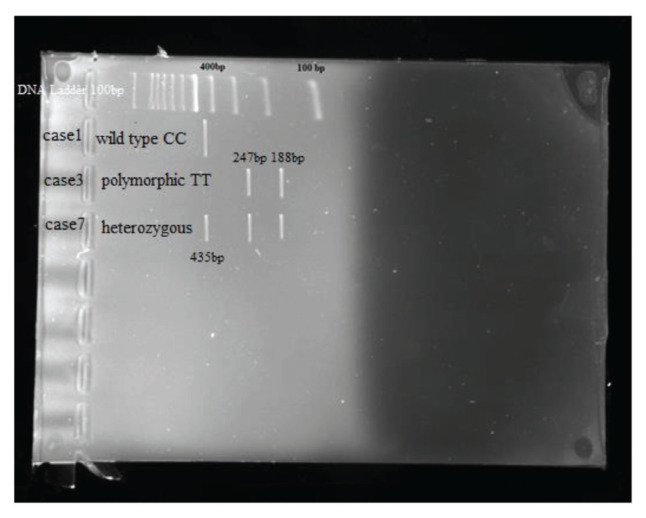
Samples of MMP-9 PCR-RFLP (435 bp) CC genotype with one fragment band, TT genotype with two fragments are polymorphic and CT genotype with three fragments band are heterozygous

The risk of ischaemic stroke associated with the heterozygous and homozygous polymorphic genotypes was done by calculation the OR using binary logistic regression analysis. The frequencies of CC genotypes versus CT genotypes (adjusted OR [aOR] = 7.09; 95% CI: 3.41, 15.38; *P* < 0.001) and CC genotypes versus TT genotypes (aOR = 19.75; 95% CI: 8.0, 56.83; *P* < 0.001) genotypes differed significantly between the two groups. aOR for the C allele versus T allele showed that the T allele of the MMP-9 gene added a 10.71-fold risk (aOR = 10.71; 95% CI: 5.65, 21.09; *P* < 0.001) for the development of ischaemic stroke in our population (Table 4, Figure 1). Likewise, a significant risk of ischaemic stroke was associated with the CT genotypes (aOR = 7.09; *P* < 0.001) and TT genotypes (aOR = 19.75; *P* < 0.001). In the cases, the allele frequency for the C allele was 43.5% and 56.5% for the T allele; whereas, among controls, it was 86.5% for the C allele and 13.5% for the T allele. Also, the T allele was associated with a significant risk of ischaemic stroke (aOR = 10.71; *P* < 0.001).

### Methylation Levels in Ischaemic Stroke and Healthy Subjects

**Figure 2:**
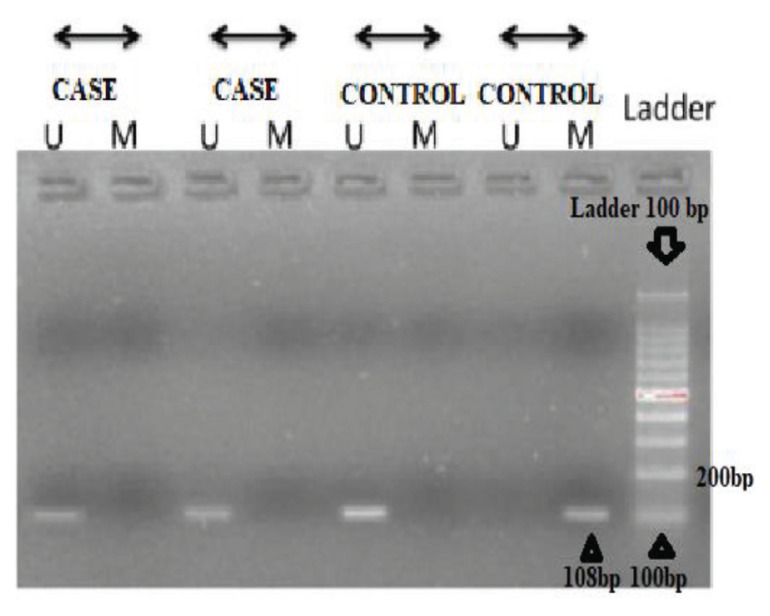
Agarose gel electrophoresis of MMP-9 gene MSP products from peripheral blood. M represents methylation specific primers; U represents unmethylated specific primers

In each group, both the methylated and unmethylated primers amplified specific PCR bands. However, there was a large difference in the methylation index. In the peripheral blood, the methylation indices were 19% and 51% for the cases and controls, respectively, and this difference was statistically significant (χ^2^ = 21.26; aOR = 0.23; 95% CI: 0.12, 0.43; *P* < 0.001) as shown in Table 5 and Figure 2. We also tried to determine if there was a correlation between MMP-9 gene polymorphism and hypermethylation in the MMP-9 gene-promoter region in the cases. As per our findings, for the CC genotype, 47 cases were methylated and 59 were unmethylated; for the CT genotype, 13 cases had methylation and 35 were unmethylated; and for the TT genotype, 9 cases had methylation and 37 controls were unmethylated.

## Discussion

Stroke is the second leading cause of death worldwide and is associated with significant morbidity and mortality. While multiple risk factors have been identified for stroke, the aetiology remains unexplained in many patients. In the present study, we tried to explore the association between MMP-9 polymorphism and methylation in ischaemic stroke patients.

The anti-atherogenic potential of high density lipoprotein (HDL) is due to its heterogeneous shape, size and density. In our study, the mean serum HDL level was significantly lower in cases 35.3 (14.24) mg/dL as compared to controls 39.2 (7.95) mg/dL (*P* = 0.019).

Sacco et al. (25) also reported similar findings that the total HDL was significantly lower in patients with stroke than controls. While the role of raised total cholesterol (TC) levels in stroke remains controversial, its role in coronary artery disease is an established phenomenon (26). Although observational studies have not pointed out a clear association between the TC levels and stroke, few other studies have reported an association between high serum TC levels and increased risk of ischaemic stroke (27, 28). In our study, the mean serum TC was 143.25 mg/dL in the cases and 116.69 mg/dL in the controls (*P* < 0.001); however, these values were within the desired range. Also, the mean serum triglyceride (TG) level was 130.67 mg/dL for the cases and 142.68 mg/dL for the controls. This finding is contradictory to the previous studies which indicated a temporal relationship between the serum TG level and the risk of stroke. However, this paradox of serum TG has been reported with ischaemic stroke and low levels of serum triglyceride are associated with a poor prognosis for stroke. Notably, few studies have also reported that no relationship exists between hypertriglyceridemia and the risk of stroke (29, 30).

The manifestation of stroke depends upon the area of the brain involved. In our case, right middle cerebral artery territory ischaemic infarct was most commonly seen on CT imaging, which coincides with the findings of other studies that reported the right middle cerebral region ischaemic infarct as the most common type of ischaemic infarct (31). Other common stroke types observed in our cases were left frontoparietal region infarct and left middle cerebral artery territory infarct.

The ECG abnormalities in stroke vary from T-wave abnormalities, arrhythmias, QT-interval prolongation to atrial fibrillation (AF) causing a five-fold increase in the incidence of stroke (32). In our study, AF was seen in 22% of cases. AF induces blood stasis leading to thrombus formation and embolism to the brain. Likewise, arrhythmia is still considered a primary cause of thromboembolism, along with other abnormalities. In our cases, 61% of patients had an NSR, while T-inversion in the anterior, inferior and lateral chest leads were seen 5%, 3%, and 3% of cases, respectively.

It is known that approximately 20% of stroke has an atherosclerotic aetiology (33). Carotid Doppler is performed routinely in stroke patients to evaluate the status of extracranial arteries supplying the brain. An elevated risk of large-artery stroke is observed in patients with the highest degree of carotid stenosis along with other factors. In our cases, 14% of stroke patients had near-total occlusion of bilateral distal right carotid arteries, while 37% of cases had a normal carotid doppler, indicating multifactorial aetiology of stroke.

This study focused on the genetics of the MMP-9 gene polymorphisms and their association with ischaemic stroke. Our results revealed that in the case of MMP-9 gene SNP rs3918242 (C→T), subjects with the CT genotypes had 7.09 times higher risk of developing ischaemic stroke compared to the CC genotype. Also, the subjects with the TT genotype had 19.75 times higher risk of developing ischaemic stroke as compared to the CC genotype. A previous study by Buraczynska et al. (17) also showed a silent C-to-T substitution associated with ischaemic stroke in a study comprising of 322 cases and 410 controls. However, replication of this association has been highly variable, as another study by Kaplan et al. (19) found no association of MMP-9 haplotype in causing ischaemic stroke. In the same study, another molecule, the MMP-3 haplotype 2, was associated with a reduced risk of myocardial infarction and increased risk of haemorrhagic stroke while showing no risk for ischaemic stroke. Another study in the Chinese population by Zhao et al. (18), which studied MMP-3 and MMP-9 gene polymorphism, revealed that SNP rs3918242 of MMP-9 gene was associated with an increased risk of ischaemic stroke, (OR = 5.47) similar to our results.

The epigenetic state is closely linked to the causation of stroke. DNA-methylation, an epigenetic modification, represses global and genetic transcription affecting neuronal and glial development (34). Higher levels of methylation cause gene silencing and consequently, its expression. The differential expression of DNA-methylation depends upon the differentiation of progenitor cells into specific neural lineages. Loss of genomic DNA-methylation has been seen in many diseases including stroke. Whereas, MMP-9 gene-promoter region hypermethylation leads to silencing of the gene and decreases the risk of ischaemic stroke. Therefore, detection of MMP-9 gene methylation in ischaemic stroke patients may serve as a novel approach to predict the risk of stroke. Promoter region hypomethylation and its role in the development of stroke and identifying methylated areas among CpG islands are potential biological markers for early diagnosis. This is of paramount importance in stroke as predicting genetic susceptibility to stroke can increase the chances of early detection and increased survival. Several studies have revealed the role of DNA-methylation in stroke in the subset of patients with no history of comorbidities. These sporadic cases manifest ischaemic stroke at an early age; moreover, hypomethylation increases the chances of conversion of an ischaemic infarct into a haemorrhagic one, leading to significant mortality and morbidity (35).

Our study furthers the previous research on MMP-9 gene methylation, which is consistent with the observation that MMP-9 gene methylation is a relatively frequent event in sporadic ischaemic stroke and promoter methylation of MMP-9 gene plays a pivotal role in functional inactivation. We also investigated MMP-9 gene-promoter methylation in both cases and control groups, which revealed that methylation was seen in 51% of controls, while only 19% of cases had methylation. Methylation was not detected in 81% of cases and 49% of controls (*P* < 0.001) which was statistically significant, similar to Lin (35), who reported significant MMP-2 methylation levels in a subgroup of stroke patients, with a significant association in men. Another study by Baccarelli (23) involving 712 elderly subjects described the differences in a repetitive element of DNA methylation for predicting the risk of developing stroke. The associations of long interspersed element-1 (LINE-1) hypomethylation and stroke suggest that DNA-hypomethylation anticipates disease diagnosis and that hypomethylation may help in identifying the individuals at risk before the onset of clinical disease. We observed similar findings that hypomethylation was associated with an increased risk of stroke. Another study by Soriano-Tárraga et al. (24) involving 281 ischaemic stroke patients and 99 controls stated that global DNA-methylation was not associated with ischaemic stroke subtypes in either cohort or as a continuous variable or in quartile categories of the Luminometric Methylation assay (LUMA) in the study. On the contrary, we found a temporal relationship between the incidence of stroke and hypomethylation. Several factors contribute to the differential expression of the frequency of methylation which may be due to exposure of the study population to specific environmental agents or the MSP detecting differential methylation status by bisulphite-treated DNA with primers specific for methylated and unmethylated DNA.

There were certain limitations in our study. Ischaemic stroke was excluded in control subjects only by medical history and a lack of neurological manifestation; however, ideally, the possibility of controls having a silent stroke could not be ruled out without brain imaging. Secondly, the sample size was small and could not represent the whole of North-Indian population. Future research is needed to establish a concrete relationship of genetics with stroke among the Indian population.

## Conclusion

The TT and CT genotypes of the MMP-9 gene-1562 C/T polymorphism (SNP rs3918242) and its hypomethylation increase the individual’s susceptibility to stroke in the Indian population and the MMP-9 gene may be considered as a candidate gene for determining predisposition to ischaemic stroke. Consequently, MMP inhibitors could serve as a therapeutic modality to provide a genetic basis for stroke prevention.

## Figures and Tables

**Table 1:** Comparison of biochemical parameters among cases and controls

Variables	Mean (SD) for cases	Mean (SD) for controls	Mean difference (95% CI)	*t*-statistic (df)	*P-*value
Serum urea (mg/dL)	49.31 (43.78)	33.67 (13.28)	−15.64 (−24.66, −6.61)	−3.41 (198)	0.001
Serum creatinine (mg/dL)	1.16 (1.03)	0.74 (0.29)	−0.42 (−0.64, −0.21)	−4.00 (198)	< 0.001
Total serum bilirubin (mg/dL)	0.673 (0.53)	0.71 (0.34)	0.03 (−0.09, 0.15)	0.50 (198)	0.612
Serum AST (IU/L)	52.13 (91.89)	46.80 (30.70)	−5.33 (−24.43, 13.77)	−0.55(198)	0.583
Serum ALT (IU/L)	42.37 (97.31)	55.28 (61.02)	12.91 (−9.74, 35.56)	1.12(198)	0.262
Serum ALP (IU/L)	142.84 (134.11)	55.71 (61.10)	−87.13 (−116.19, −58.06)	−5.91(198)	< 0.001
Serum TG (mg/dL)	130.67 (38.1)	142.68 (25.54)	12.01 (2.96, 21.05)	2.61(198)	0.010
Serum total cholesterol (mg/dL)	143.25 (67.29)	116.69 (29.97)	−26.56 (−41.08, −12.03)	−3.60(198)	< 0.001
Serum HDL (mg/dL)	35.34 (14.24)	39.20 (7.95)	3.86 (0.64, 7.07)	2.36(198)	0.019
Serum LDL (mg/dL)	96.40 (17.88)	84.39 (33.25)	−12.01 (−19.46, −4.56)	−3.18(198)	0.002

**Table 2:** Distribution of MMP-9 genotypes and allele frequencies of the study groups

MMP-9 gene-1562C/T polymorphism (SNP rs3918242)	Case (*n* = 100)	Controls (*n* = 100)
CC genotype	26%	79%
CT genotype	35%	15%
TT genotype	39%	6%
C allele	43.5 %	86.5%
T allele	56.5%	13.5%

**Table 3:** Genotype distribution of MMP-9 gene and Chi-squared values among cases and controls

MMP-9 gene-1562C/T polymorphism (SNP rs3918242)	Case (*n* = 100)	Control (*n* = 100)	df	χ^2^	*P-*value
CC	26%	79%			
CT	35%	15%	2	58.95	< 0.001
TT	39%	6%			

**Table 4:** Asociation of MMP-9 gene SNPs with the risk of ischaemic stroke

Dependent: disease		Absent	Present	OR (Binary logistic regression)
SNP rs3918242	CC	79 (75.2)	26 (24.8)	-
	CT	15 (30.0)	35 (70.0)	7.09 (3.41, 15.38, *P* < 0.001)
	TT	6 (13.3)	39 (86.7)	19.75 (8.00, 56.83, *P* < 0.001)
C allele	Absent	6 (13.3)	39 (86.7)	-
	Present	94 (60.6)	61 (39.4)	0.10 (0.04, 0.23, *P* < 0.001)
T allele	Absent	79 (75.2)	26 (24.8)	-
	Present	21 (22.1)	74 (77.9)	10.71 (5.65, 21.09, *P* < 0.001)

*Note: P*-value < 0.05 = significant

**Table 5:** Comparison of MMP-9 gene methylation in study group

Group	Methylated	Unmethylated	OR (95% CI)	χ^2^	*P*-value
Case	19	81	0.23 (0.12, 0.43)	21.26	< 0.001
Control	51	49			
